# Random optical parametric oscillator fibre sensor

**DOI:** 10.1038/s41377-025-02049-9

**Published:** 2026-01-04

**Authors:** Pedro Tovar, Jean Pierre von der Weid, Yuan Wang, Liang Chen, Xiaoyi Bao

**Affiliations:** 1 Nexus for Quantum Technologies, University of Ottawa, Ottawa Rio de Janeiro, ON Canada; 2 Department of Electrical Engineering, Pontifical Catholic University of Rio de Janeiro, Rio de Janeiro, RJ Brazil

**Keywords:** Imaging and sensing, Optical sensors, Fibre optics and optical communications

## Abstract

Fibre laser-sensors have emerged as a promising solution for long-distance sensing, offering high SNR and fine spatial resolution. However, their adoption is constrained by fundamental limitations: they typically require a fixed mirror at the sensing location or access to both fibre ends for electronic selection of the sensing location. This work introduces a random optical parametric oscillator (R-OPO) fibre sensor that addresses these challenges. Similar to a laser-sensor but exploiting modulation instability and continuous weak reflections, the R-OPO sensor enables long-distance access ( > 25 km) sensing by arbitrarily addressing 1 m-long fibre sections over a long sensing range ( > 1 km). It supports both backward and forward sensing, but unlike most forward sensors, the sensing location information is readily available at both fibre ends. Most importantly, it eliminates the need for a fixed mirror at the sensing location, offering electronically tunable sensing locations. The proposed detection scheme enables straightforward quantitative measurement of dynamic perturbations, requiring only a single fast Fourier transform, thus enabling real-time monitoring. Temperature and strain noise-limited sensitivities of 10.73 ${\mu }^{o}C/\sqrt{Hz}$ and 80.6 $p\varepsilon /\sqrt{Hz}$ were obtained. Taking advantage of four-wave-mixing by-products inherent to R-OPOs, the sensitivity to external perturbations could be enhanced by a factor of two compared to conventional Rayleigh-based sensors. A simple frequency-unwrapping algorithm is proposed to extend the dynamic measurement range, and the continuous monitoring of a 2 °C temperature increase was accurately measured. This first demonstration of a R-OPO fibre sensor establishes the foundations for parametric fibre sensors.

## Introduction

Sensing physical quantities by means of light propagation in optical fibres have been triggering scientists for several years now, with a number of novel techniques being reported every year^1^. In most research works, one of two well-known approaches is employed: point or distributed sensing. The former is usually realised with fibre Bragg gratings (FBG) inscribed in single-mode fibres (SMF). Due to their high reflectivity, FBGs can be used for single-end ultra-long distance sensing, as their strong reflection translates into a detection with high signal-to-noise ratio (SNR). Point-sensors naturally offer high spatial resolution as the sensing element is usually in the centimetre/millimetre scale. These features make FBG-based sensors highly reliable, to the extent that they have been seamlessly integrated into diverse commercial products, finding applications in the aviation industry^2^, oil and gas pipeline monitoring^3^, civil structure vibration supervision^4^, train track monitoring^5^, among many others. However, point-sensing systems have several limitations. They are highly inflexible in terms of sensing-points location; once the sensing-elements are inscribed, they can no longer be changed to different positions in the fibre. Fibre grating arrays provide quasi-distributed sensing capabilities for long-haul applications. However, they are insensitive to perturbations between sensing points and involve high costs associated with both the inscription of multiple FBGs and the detection apparatus. The latter typically requires advanced techniques, such as wavelength scanning and/or time-division multiplexing, to optimize the number of FBGs within a single fibre^6,7^. Yet, these approaches often compromise the measurement range due to cross-talk between FBGs.

On the other side of the spectrum of fibre sensors are distributed sensors. In essence, distributed sensors make use of light scattering properties that are inherent to optical fibres and are present along its entire length to measure changes in the fibre’s refractive index. Rayleigh scattering, stimulated Raman scattering (SRS) and stimulated Brillouin scattering (SBS) have been intensively studied and successfully explored in sophisticated approaches for distributed sensing^8^. One distinguished technique, resorting on Rayleigh scattering, is phase-sensitive optical time domain reflectometry (Φ-OTDR), which consists in sending pulses from a coherent light source to an optical fibre and measuring the backscattered signal^9–11^. The detected trace exhibits a jagged profile, which is caused by randomly spaced SiO_2_ molecules that backscatter light in standard amorphous fibres. As the duration of the launching pulses is made much smaller than the coherence time of the light source, the backscattered light coherently adds up, producing a jagged envelope that is stable in the absence of external perturbations, but changes in the presence of acoustic vibrations. Due to the non-linear conversion of acoustic vibrations (or temperature changes) into the trace’s amplitude, a number of techniques have been proposed to quantify environmental perturbations, including coherent detection-based phase-measuring Φ-OTDR^12^, chirped-pulse Φ-OTDR^13^, wavelength-scanning coherent OTDR^14^, speckle tracking Φ-OTDR^15^ and frequency multiplexed Φ-OTDR^16^. Despite many differences, all techniques exhibit SNRs inferior to those obtained with point-based sensors. The SNR discrepancy is due to the large difference in the reflection coefficients: the Rayleigh-reflectivity of one meter of standard SMF is approximately 0.0000063%, while FBGs can be manufactured with reflectivities close to 99%.

There is a third category of fibre-optic sensors, which in the spectrum of fibre sensors lies somewhere in between point-based and distributed approaches: the electronically addressable fibre sensors (EAFS). These consist of optical systems designed to electronically select a single location in the fibre to act as a point-sensor. EAFS are particularly useful for applications that do not require continuous monitoring of the whole fibre, such as bridge monitoring, where the focus is on critical structural points. Because the sensing location can be tuned electronically, it offers far more flexibility than traditional point-based sensors. An example of EAFS technique is Brillouin optical correlation-domain analysis (BOCDA)^17,18^, involving the launch of synchronous frequency-modulated probe and pump signals in opposite directions into a fibre, with SBS taking place at a unique location at a time. By scanning the delay between frequency-modulated probe and pump, any location in the fibre may be addressed, thus enabling the sensing of the entire fibre. Its main limitation is the requirement of access to both ends of the fibre, being a significant drawback for most practical applications. Although single-end access sensing has been demonstrated with Brillouin optical correlation-domain reflectometry (BOCDR)^19^, it is limited to short distances due to the fast depletion of the Brillouin pump wave.

A single-end access EAFS based on Rayleigh scattering has been recently proposed^20^. The approach consists in synchronising the Rayleigh backscattering light from a small fibre section with a pulsed gain mechanism to form a laser-sensor. By using the Rayleigh backscattering from a small section of an SMF as one of two mirrors in a fibred cavity, where the other mirror is simply a fixed point reflector at the launching end, environmental perturbation in the synchronised fibre section directly affects the laser cavity, hence altering the properties of laser light and thus acting as a laser-sensor. The ease of sensor localisation makes laser-sensors more flexible (and cheaper) than point sensors, while their strong lasing light offers significantly higher SNR compared to distributed sensors.

Laser-sensors have also been developed based on Brillouin scattering^21^, but the same limitations of BOCDA remain. In^20^, a hybrid gain mechanism from a semiconductor optical amplifier (SOA) and an Erbium-doped fibre amplifier (EDFA) was explored to develop a Rayleigh-based laser sensor which was shown to measure vibrations at a short fibre segment. Although 5 kHz vibrations could be detected with high SNR, the system did not allow quantitative evaluation of applied vibrations, the same problem faced by Φ-OTDR systems without phase detection. In a more recent publication^22^, it was shown that the spectral line of a random-laser EAFS based on Rayleigh scattering shifts linearly with environmental perturbations, therefore allowing quantitative measurements. This linear relationship between either strain or temperature variations and optical frequency shifts is similar to spectral shifts undergone by an FBG, while in the latter the refractive index is made periodically changing, in the former it varies randomly in sub-wavelength scale. However, due to the narrow spectral envelope of the random laser-sensor, which is defined by the Fourier transform of the laser’s pulse width (50–200 MHz), spectral shifts could only be measured with the use of a high-resolution optical spectrum analyser (HR-OSA), with a resolution of 0.16 pm (or 20 MHz). Not only HR-OSAs are expensive equipment, but their slow scanning speed prevents dynamic measurements, thus limiting the spread of EAFS.

In this work, a novel EAFS is theoretically proposed and experimentally verified. In our previous work^23^, we described the physics of a coherent light oscillator named random optical parametric oscillator (R-OPO), which is enabled by modulation instability (MI) and continuous weak reflection. Taking advantage of the high and distributed amplification offered by MI, as well as the high temperature/strain sensitivity of single-mode fibres, we demonstrate here the first R-OPO fibre sensor. It offers long-distance, high-resolution and high-sensitivity sensing, with features similar to a laser-sensor. However, while in conventional laser-sensors non-linear effects are usually avoided, here they are taken advantage of, thus potentially offering the sensing at significantly longer distances. In addition, we believe this to be the first laser-like EAFS offering access to the sensing information at both fibre ends while allowing dynamic and quantitative strain/temperature measurements.

## Results

### Sensing principle

**Fig. 1:**
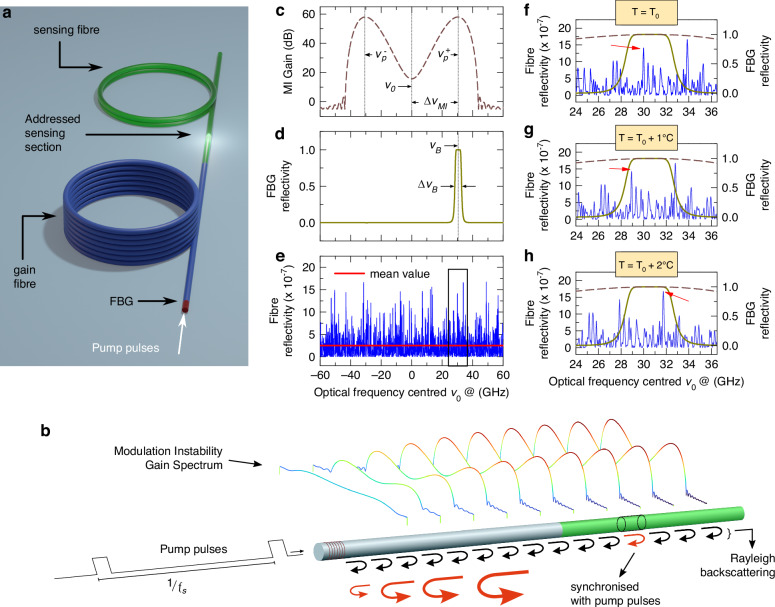
**Principle of R-OPO fibre sensor**. **a** Simplified experimental configuration. **b** Operation principle: as MI gain builds up along the fibre, MI sidebands are continuously reflected. The reflected light from a unique fibre segment synchronized with pump pulses experiences MI amplification when further reflected at the FBG, enabling parametric oscillation. **c** MI gain profile. **d** FBG reflectivity spectrum. **e** Example of simulated reflected spectrum from a 1 m-long fibre segment. (**f**–**h**) show the shift of the reflected spectrum when the temperature of the fibre segment is increased. Red arrows point to the optical frequency spike at which parametric oscillation initiates

The principle of the R-OPO fibre sensor is depicted in Fig. 1. It is composed of a high-power pulsed laser source with adjustable repetition rate, a tuneable FBG and two cascaded segments of SMF (Fig. 1a). In the experiments, we selected fibres with 25.5 km and 1 km, but as it will be clear shortly, different fibre lengths can be used. High-power pump pulses centred at *ν*_0_ with a duration of 10 ns and a repetition rate of  ~4 kHz are launched into the SMFs through the FBG, which is centred at *ν*_*B*_ and has a 3 dB-spectral width of Δ*ν*_*B*_. The FBG’s centre frequency is set to be significantly higher than *ν*_0_ to allow direct propagation of pump pulses. As the pulses propagate through the fibre, self-phase modulation (SPM) initiates governed by the Kerr effect. If the system operates under anomalous group-velocity dispersion (GVD parameter *β*_2_ < 0), modulation instability (MI) sidebands build-up around *ν*_0_ (see Fig. 1b)^24^. This non-linear effect traduces in parametric gain for wavelengths falling within the MI sidebands, with peak MI gain at frequency ${\nu }_{p}^{\pm }={\nu }_{0}\pm \Delta {\nu }_{MI}$, where  ± stands for the higher and lower frequency sidebands, and Δ*ν*_*MI*_ is the detuning frequency from *ν*_0_. The peak MI gain can be estimated by^25^:

1 $${g}_{p}=1+2\,{\sinh }^{2}(\gamma {P}_{0}L)$$

where *γ* is the non-linear coefficient due to Kerr effect, *P*_0_ is the input peak power and *L* is the fibre length. Peak gain occurs at a detuning frequency $\Delta {\nu }_{MI}={\Omega }_{c}/\sqrt{2}$, where *Ω*_*c*_ is the MI cut-off frequency given by ${(4\gamma {P}_{0}/| {\beta }_{2}| )}^{1/2}$. Figure 1c shows the theoretical MI gain profile at the end of the fibre when 10 ns-long pump pulses with 285 mW peak power are launched into a 25.5 km-long fibre, resulting in Δ*ν*_*MI*_ ≈ 30 GHz (see simulation details in Supplementary Notes).

As the optical pulses propagate into the fibre, photons are continuously backscattered at both *ν*_0_ and at the MI sidebands. By tuning the FBG centre frequency to the peak of one of the MI sidebands (*ν*_*B*_ = *ν*_0_ + Δ*ν*_*MI*_), as shown in Fig. 1d, then the Rayleigh backscattered light from that MI sideband (MI-Rayleigh light) is reflected at the FBG and relaunched into the fibre. Clearly, since the Rayleigh reflectivity is extremely small, the MI-Rayleigh light reflected at the FBG does not continuously circulate in the fibre; it rather vanishes when no optical gain is present. To enable its circulation, i.e., to achieve optical oscillation, the repetition rate of pump pulses (4 kHz) is chosen such that the Rayleigh backscattered light from a fibre segment with 1 m length (10 ns pulse width) located at the 1 km-long fibre is synchronised with pump pulses. Hence, MI-Rayleigh light from the addressed fibre segment back-propagates and reaches the FBG together with the sub-sequent incoming pump pulse. In such a way, the MI-Rayleigh light acts as a seed for MI gain, experiencing parametric amplification as it co-propagates with pump pulses. By fine-tuning the repetition rate of pump pulses, any 1 m-long fibre segment within the 1 km fibre may be selected as the Rayleigh feedback segment; this feature makes it an ideal platform for a single-end access EAFS as will be clear soon. Note that, for pulses with a duration of 10 ns, dispersion effects are negligible regarding the co-propagation of pump pulses and MI-Rayleigh light. It follows that MI-Rayleigh light experiences strong amplification along the first fibre, thus working as a gain fibre.

The second fibre acts as a sensing fibre. In case a standard SMF were used as the sensing fibre, then the reflection losses from a 1 m-long segment would be in the order of −72 dB, which, taking into account the propagation losses of a round-trip length of 51 km, would result in a total round-trip loss of about −82 dB (assuming an FBG with reflectivity 0.99). If the MI-gain is sufficient to compensate the round-trip losses, then parametric oscillation would initiate. The threshold condition for parametric oscillation is written as:

2 $${g}_{p}({z}_{s},\nu )\,\max [{R}_{fibre}(\nu ,{z}_{s})]\,{R}_{FBG}(\nu ) > 1$$

where *g*_*p*_ is the accumulated parametric gain at position *z*_*s*_, which is the fibre location synchronised with pump pulses. *R*_*FGB*_ is the FBG’s reflectivity, and *R*_*fibre*_ corresponds to the reflectivity of Rayleigh backscattering in the fibre. Its average value, 〈*R*_*fibre*_〉, is the reflection loss that one would measure in a power meter or in a conventional OTDR, which changes minimally for adjacent short fibre segments (0.2 dB/km), but the spectrum of *R*_*fibre*_(*ν*, *z*_*s*_) exhibits strong fluctuations^26^. This feature can be easily observed in time-domain in Φ-OTDR traces. The interference of backscattered light with random phases within half the pulse width for a given location *z*_*s*_ results in constructive or destructive interference (or any possibility in between), producing the well-known jagged OTDR traces. It turns out that the interference outcome is highly frequency-dependent for any location in the fibre^27^, where the correlation between neighbouring frequencies is defined by the inverse of the pulse width^28^. A realisation of the reflectivity spectrum using the theoretical model from^29^ is presented in Fig. 1e, where a pulse width of 10 ns was used.

Eq. (2) can be analysed graphically as shown in Fig. 1f, where *R*_*fibre*_ and *R*_*FBG*_ are presented, and the profile of *g*_*p*_ is shown as a reference (dashed line). If sufficient MI-gain is available, then parametric oscillation would preferably start at the optical frequency of highest *R*_*fibre*_ within the FBG bandwidth (indicated by a red arrow). Due to the randomness of the Rayleigh reflectivity spectrum, this type of parametric oscillator is referred to as Random Optical Parametric Oscillator (R-OPO)^23^. One of the main concerns to reach parametric oscillation in this configuration is the pump power, which cannot be arbitrarily increased to enhance the MI-gain as other non-linear effects start to take place at higher powers. We showed in^23^ that three approaches can be explored to reduce the total losses and achieve random parametric oscillation: 1) increasing the duration of pump pulses so that more light is backscattered for each pulse; 2) increasing the number of addressed sections by increasing the pulse repetition rate, resulting in a large number of fibre sections synchronised with pump pulses; or 3) by using an enhanced optical fibre designed for sensing applications^30^, which offers 16 dB more reflectivity than Rayleigh scattering. This is achieved by engineering fibres with ultra-weak continuous random Bragg gratings, offering directional reflection rather than omnidirectional scattering, thus also preserving low attenuation in the forward direction^31^. This third approach was used in the simulation of the reflectivity spectrum of a 1 m-long fibre segment in the sensing fibre shown in Fig. 1e, indicating a mean reflectivity of  −82 + 16 = −66 dB ( ~2.5 × 10^−7^), and a peak reflectivity of  −57.5 dB. Within an optical bandwidth of about Δ*ν*_*B*_ = 3 GHz, and for a 1 m-long fibre section, the peak reflectivity is nearly 10 dB higher than 〈*R*_*fibre*_〉^23^, enabling the onset of parametric oscillation at lower pump powers and at a narrow spectral line (*ν*_*R*−*OPO*_). For the simulated case shown in Fig. 1f, the round-trip loss in the oscillator is  ~ 57.5 dB, which can be compensated by the peak MI gain ( ~58 dB) accumulated over 25.5 km for pump pulses with a peak power of 285 mW (see calculation details in Supplementary Notes), thus enabling the onset of random parametric oscillation.

The approach 1) described above brings two problems. First, pulses longer than  ~10 ns compromise MI gain as other non-linear effects such as SBS and SRS are promoted^32^. And second, longer pulses reduce the spatial resolution of the R-OPO sensor, which is obviously undesired and discarded for high-resolution sensing applications. Although the approach 2) may be more convenient than 3) as standard SMF are vastly available, the R-OPO emission spectrum depends on the interference of light between all the synchronised sections. Hence, random shifts on the R-OPO emission frequency are continuously observed^23^, caused by environmental fluctuations all along the fibre. In the third approach, the R-OPO emission frequency may be selected from the Rayleigh reflectivity spectrum exclusively from one fibre section synchronised with pump pulses. However, to ensure that only one fibre section is synchronised, the following condition must be satisfied:

3 $$\left\lfloor 2L{f}_{s}/{v}_{g}\right\rfloor =1$$

where *L* is the total fibre length equal to *L*_*g*_ + *L*_*s*_ (lengths of the gain and sensing fibres), *f*_*s*_ is the pulse repetition frequency, *v*_*g*_ is the group velocity, and $\left\lfloor \cdot \right\rfloor$ is the round-down operator. The left side of Eq. (3) gives the number of fibre sections synchronised with pump pulses^23^, which must be equal to one. Indeed, this is a limitation of the R-OPO sensor, as segments in the first half of the total fibre length may not be addressed. In other words, since the addressed segment must be somewhere along the sensing fibre, it is required that:

4 $${L}_{g}\ge {L}_{s}$$

Since the majority of remote fibre sensing applications require a launch fibre to connect the sensing apparatus at a measurement station to the sensing fibre, and given that the launch fibre works as the gain fibre, Eq. (4) can be easily satisfied, thereby enabling the sensing of any section along the sensing fibre.

With only one fibre section synchronised with pump pulses, any spectral shift (Δ*ν*) in the R-OPO emission frequency can be directly associated to temperature or strain variations at the addressed section through^33^:

5 $$\frac{\Delta \nu }{{\nu }_{0}}\approx -0.78\,\Delta \varepsilon $$

6 $$\frac{\Delta \nu }{{\nu }_{0}}\approx -(6.92\times 1{0}^{-6})\,\Delta T$$

where *ν*_0_ is the R-OPO emission frequency, and strain/temperature variations are represented by Δ*ε* and Δ*T*, respectively. Note that, although Eqs. (5) and (6) are well known for their relevance in Rayleigh-based fibre sensors, there is a particular difference between R-OPO frequency shifts and spectral shifts from conventional fibre sensors. For instance, as shown in^33^ for Φ-OTDR systems, Eqs. (5) and (6) are used to determine how much the probe laser frequency would have to shift to account for the observed changes in the trace envelope. Thus, the probe laser frequency does not change, and in fact, it must be highly stable to prevent false strain/temperature readings. On the other hand, the R-OPO frequency actually changes under environmental perturbations as discussed next.

Assuming that the Rayleigh reflectivity spectrum in Fig. 1f was obtained at a temperature *T*_0_, a temperature increase of 1°C at the 1 m-long section would cause a spectral shift as shown in Fig. 1g. This spectral shift means that the coherence sum of the reflected signal along the addressed fibre section is highest at a new wavelength within the FBG bandwidth, so that parametric oscillation is now sustained at a new optical frequency. Note that, for the particular Rayleigh spectrum presented, which is the result of a random realisation of refractive indexes along the addressed fibre section, the increase of one degree Celsius simply shifts the highest reflectivity peak to a lower frequency, but the same peak is the highest within the FBG bandwidth, thus still selected for parametric oscillation. When temperature is increased by another degree, a new reflectivity peak becomes the highest (pointed by a red arrow in Fig. 1h). In that case, the R-OPO exhibits mode-hopping. In the following sections we will show that the spectral shifts in the R-OPO oscillating line can be accurately measured in a straightforward way, without the need of a high-resolution OSA, enabling dynamic temperature and strain monitoring either with or without mode hopping, allowing high SNR measurements with ultra-high sensitivity for long sensing distances, while offering high spatial resolution, given by the width of the pump pulse.

### Dynamic temperature sensing

**Fig. 2:**
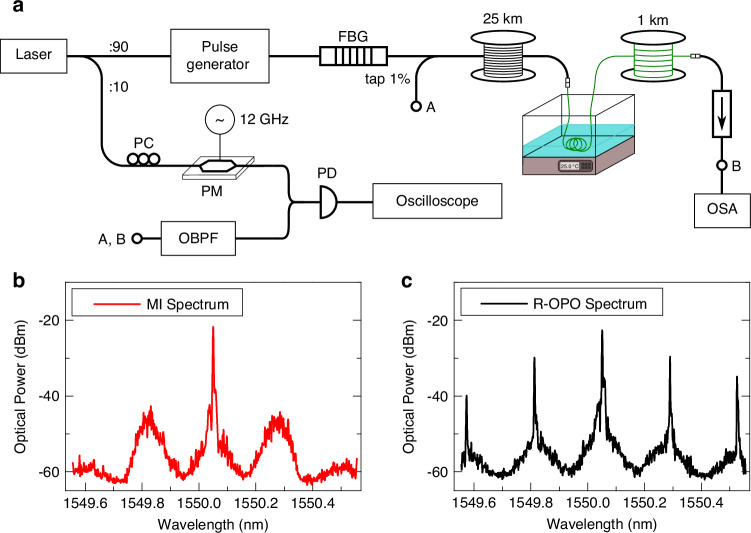
**Experimental setup and optical spectra**. **a** R-OPO experimental setup for temperature sensing. Optical spectra measured at point *B* below and above the oscillation threshold are shown in (**b**, **c**), respectively

The detailed experimental setup of the R-OPO fibre sensor is displayed in Fig. 2a. A narrow linewidth (Δ*ν*= 5 kHz) continuous wave (CW) laser source at 1550.05 nm is split in two branches, where 90% of the light is directed to the R-OPO sensor and 10% is used as reference. The former is pulsed with two cascaded electro-optic modulators (see Methods), amplified with EDFAs, and launched through an FBG centred at 1550.28 nm (Δ*ν*_*B*_ = 3 GHz) to the cascaded fibres, where a 1% tapping point is placed in between the FBG and the gain fibre. A 1 m-long fibre segment from the beginning of the sensing fibre is submerged in a water bath with controlled temperature. The repetition rate of pump pulses is fine tuned to 4,052.83 Hz to address the fibre segment under the water bath at 25,564.6 m. A polarization controller (PC) is included just before the FBG to align the polarization of light reflected from the addressed section with that of incoming pulses in order to maximize the polarization-dependent gain^34^. An isolator is placed at the end of the sensing fibre to prevent point reflections. Because the R-OPO light circulates in the half-open cavity, it can be conveniently measured either at the launching end (point *A*) or after the isolator (point *B*). Since MI amplification takes place only in the forward direction, the R-OPO light is stronger at point *B*, but the same spectrum shape is measured at both ends. Two examples of optical spectra measured at point *B*, below (pump power of 250 mW) and above (300 mW) the oscillation threshold power, are shown in Fig. 2b, c. As previously reported^23^, as soon as parametric oscillation initiates, four-wave mixing (FWM) between the pump wavelength and the R-OPO oscillating line starts, giving rise to multiple equally spaced spectral spikes.

For dynamic temperature sensing, it is desired to measure frequency shifts in the R-OPO oscillating line (see Fig. 1f–h) while avoiding long scanning times common in most spectral measurements. This could be achieved by filtering the R-OPO output with an optical band-pass filter (OBPF) to select only the oscillating line at  ~ *ν*_*B*_, and mixing it with the 1% reference light. In this case, each R-OPO pulse would carry the beat frequency *ν*_*beat*_ at around 30 GHz, which shifts when the temperature of the addressed fibre segment changes. Hence, the temperature sampling rate depends on the repetition rate of pump pulses. It turns out that the pulse repetition rate is a key sensing parameter, which at the same time limits the temperature sensing rate and defines the location of the fibre segment where temperature is sensed. Segments at the beginning of the sensing fibre are, therefore, sampled at higher rates compared to those at its end. This is different from conventional distributed fibre sensors, which have the same sampling rate for all fibre segments – due to the necessity of awaiting the return of backscattered light from the distal end of the fibre. Here, respecting the condition expressed in Eq. (4), up to two times higher sampling rates could be achieved depending on the addressed location. For instance, in the particular case where *L*_*g*_ = *L*_*s*_, and addressing the first fibre segment at the beginning of the sensing fibre, the sampling rate would be approximately *v*_*g*_/*L*_*g*_. This results in a sampling rate that is twice as high as that achievable with a conventional Φ-OTDR system, which is limited to repetition rates below *v*_*g*_/(*L*_*g*_ + *L*_*s*_). This twofold improvement represents the maximum advantage over Φ-OTDR systems. However, as the addressed section approaches the end of the sensing fibre (*L*_*s*_), this advantage diminishes, eventually reducing to a factor of one for the final fibre segment.

**Fig. 3:**
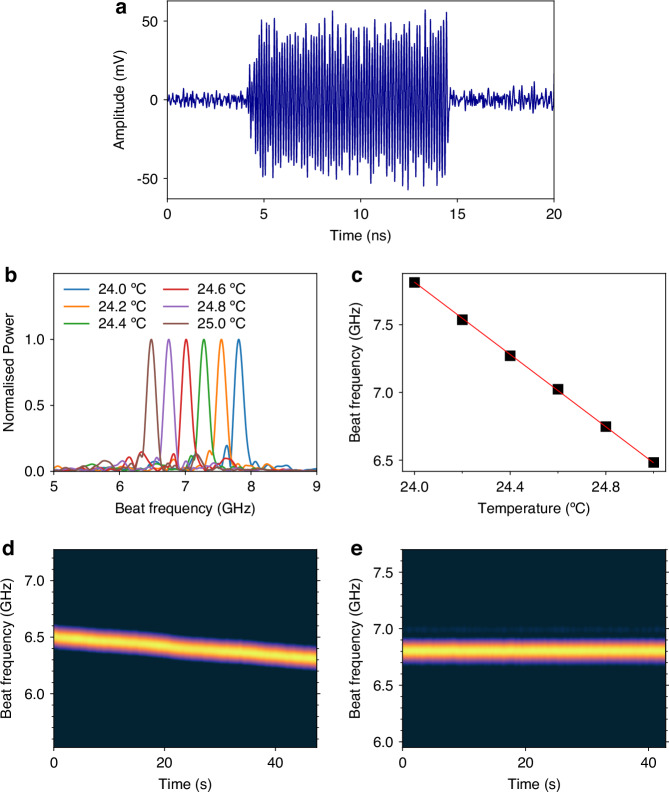
**Random OPO pulses and temperature sensing**. An example of the beating between an R-OPO pulse with the reference light is shown in (**a**). The spectrum of detected pulses shifts with temperature changes surrounding the addressed fibre segment (**b**), where a linear correspondence between temperature changes and beat frequency shift is observed (**c**). The experimental setup allows the dynamic sensing of temperature changes; by stacking the pulses’ spectra over time, the continuous monitoring of temperature changes is obtained for the segment under water (**d**) or any other segment along the sensing fibre (**e**)

While the measurement of a 10 ns pulse carrying a beat frequency tone around 30 GHz is experimentally realisable, it is preferable to measure lower frequency tones to enable the use of more affordable off-the-shelf components for detection. To reduce *ν*_*beat*_ to lower frequencies, the reference light had its polarization adjusted with a polarization controller and its frequency shifted with a phase modulator (PM), which is driven by a 12 GHz radio-frequency (RF) tone. The second-order sideband after phase modulation at *ν*_0_ + 24 GHz is then used as reference, resulting in beating tone in the range from 5 to 8 GHz, which is limited by the FBG bandwidth. Clearly, if a 24 GHz RF tone were available, as well as a 24 GHz-bandwidth PM, the first order sideband could be used. Note that *ν*_*beat*_ cannot be arbitrarily reduced by increasing the PM driving frequency: to accurately measure *ν*_*beat*_ at least 5 cycles should be present within the pulse duration of 10 ns, so that beat frequencies beyond 500 MHz are required. A PD and an oscilloscope, both with 12 GHz bandwidth, were used to capture the mixed signal, and an example of detected pulse is shown in Fig. 3a. In this example, detection was made from point *B*, but similar pulses were observed when collecting R-OPO pulses from point *A* – an EDFA was employed to provide further amplification before combining with the reference light.

The water bath temperature was increased from 24 °C to 25 °C in steps of 0.2 °C while capturing the mixing between R-OPO pulses and the PM-shifted reference. The fast Fourier transform (FFT) of pulses captured at different temperatures is shown in Fig. 3b, clearly indicating a peak at *ν*_*beat*_, which shifted with temperature. By plotting *ν*_*beat*_ against the set temperature, a linear behaviour is observed, yielding a temperature coefficient of −1.33 GHz/°C. Due to parametric oscillation, temperature of a 1 m-long fibre section is measured beyond 25.5 km with an SNR higher than 15 dB, which would not be possible by direct detection of the backscattered light, thus being a key advantage of laser-like fibre sensors.

To test the dynamic sensing capabilities of the R-OPO sensor, pulses were continuously captured over 47 s while the water temperature increased from 25 °C to  ~25.3 °C. This temperature increase was measured with the readout thermometer available in the controlled water bath display, which has a precision of 1 decimal place. The FFT of captured pulses is stacked horizontally over the measurement time as shown in Fig. 3d, indicating a total frequency shift of 0.2 GHz, corresponding to a temperature increase of 0.26 °C. This experiment was repeated for a different repetition rate, now set to 4,052.04 kHz, corresponding to the synchronisation of pump pulses with the MI-Rayleigh light from a fibre segment out of the water bath, 5 m after the previous one. The result is shown in Fig. 3e, where a stable beat frequency is observed. Note that, even though the heated fibre segment is part of the optical path of recirculating R-OPO pulses, it does not affect the R-OPO oscillating line.

To have temperature variations before the addressed segment disturbing parametric oscillation, they must be sufficiently high to change the optical path such that the same pulse repetition rate addresses a new fibre segment. For example, considering a typical thermo-optic coefficient of 10^−5^/°C, then a temperature increase of 1 °C affecting 1 m of fibre before an addressed section out of the water bath shifts the addressed segment by 10^−5^ m, which is negligible compared to the size of the addressed section, thus the same *R*_*fibre*_(*ν*, *z*_*s*_) governs the selection of the oscillating wavelength.

On the other hand, temperature variations occurring uniformly over the entire 25.5 km-long gain fibre may significantly affect the location of the addressed fibre segment. For instance, if the temperature increases uniformly by 4 °C across the gain fibre, and assuming a fixed pulse repetition rate, then a completely new fibre segment would be addressed, shifted from the previous one by 1 m. Certainly, this would pose a challenge for continuous long-term temperature monitoring, as natural day-night temperature variations along a deployed fibre could exceed 4 °C. However, this limitation can be circumvented by implementing a suitable measurement protocol. The monitoring of the addressed segment of interest could be periodically interrupted (e.g., every 10 min) to slowly scan the repetition rate of pump pulses in order to find the highest repetition rate that still supports parametric oscillation. This rate, ${f}_{{s}_{\max }}$, corresponds to the addressing of the first metre of the sensing fibre – with enhanced reflectivity. If the temperature along the gain fibre varied uniformly, then ${f}_{{s}_{\max }}$ changes proportionally, so that it can be used to track the beginning of the sensing fibre and to adjust the repetition rate for the segment of interest, thus fully compensating for uniform temperature changes along the gain fibre.

**Fig. 4:**
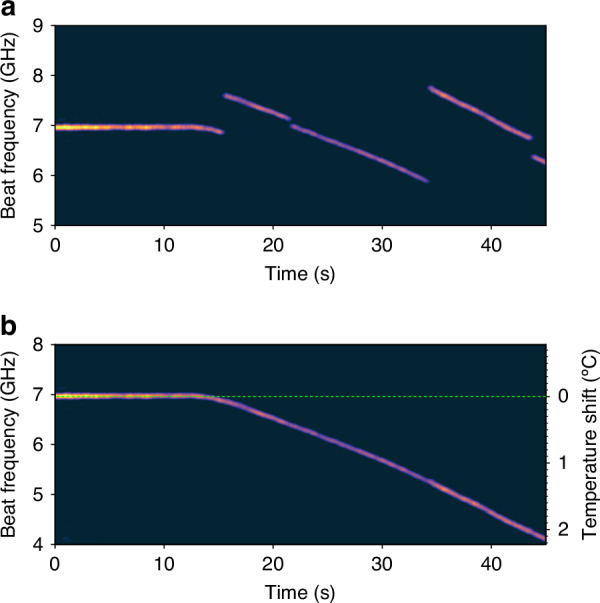
**Enhancing the dynamic measurement range**. The continuous monitoring of temperature changes surrounding a fibre segment under a water bath is shown in (**a**). A number of R-OPO mode hopping events are observed over 45 s. A frequency-unwrapping algorithm (see Methods) is proposed, allowing the extension of the dynamic measurement range, and a temperature change of 2.15°C could be directly observed (**b**)

The dynamic measurement range of temperature changes is investigated next. First, the repetition rate was reset to 4052.83 kHz to address the fibre segment under the water bath. Then, the capturing of R-OPO pulses started at time *t* = 0 s, while at the same time the water bath temperature was set to 10 °C above the current temperature of 24.6 °C. The spectrum of sequential detected pulses is shown in Fig. 4a. The thermal inertia effect of the heated volume of water is observed over the first 15 s, when the beat frequency remains stable at around 7 GHz. With the temperature increase, the beat frequency starts to drift, and eventually mode hopping occurs. Similar to phase unwrapping techniques, a simple frequency-unwrapping algorithm (see Methods) can be employed to remove the beat frequency jumps observed when the R-OPO oscillating mode hops to a new one. The frequency unwrapped result is shown in Fig. 4b, where a total temperature shift of 2.15 °C is observed over 45 s, matching with the water bath readout thermometer of 2.2 °C. For slow varying quantities such as temperature, this technique allows arbitrary enhancement of the dynamic measurement range, while the enhancement for rapidly varying quantities is limited by the sampling rate as will be discussed in the next section.

### Dynamic strain sensing

**Fig. 5:**
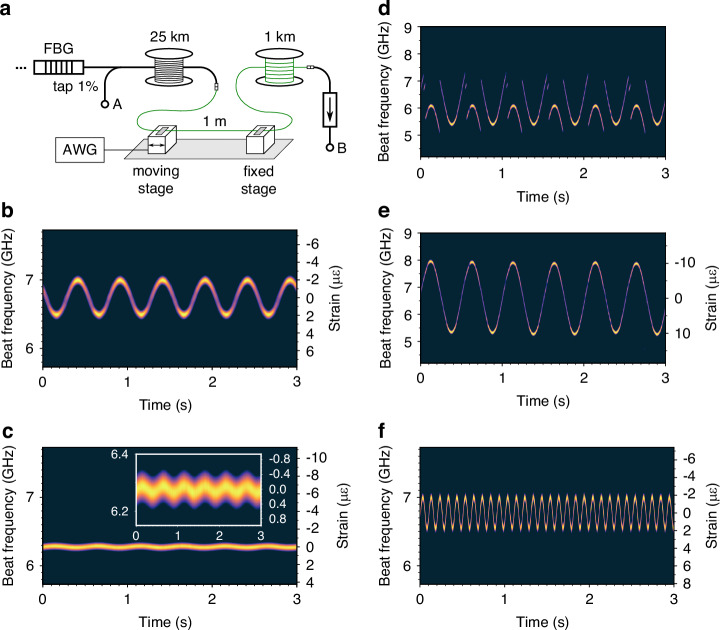
**Dynamic strain sensing**. **a** One meter from the beginning of the sensing fibre is fixed onto controllable translation stages for strain sensing. The spectrum of R-OPO pulses is shown for different configurations of applied strain:  ± 2 *με* at 2 Hz (**b**);  ± 200 n*ε* at 2 Hz (**c**);  ± 10 *με* at 2 Hz (**d**); and  ± 2 *με* at 10 Hz (**f**). A frequency-unwrapping algorithm (see Methods) is applied to the spectrogram in (**d**) to remove frequency jump errors and extend the dynamic measurement range, and its result is presented in (**e**)

Investigation of the R-OPO response to dynamic strain was conducted as follows. The 1 m-long fibre segment at the beginning of the sensing fibre was removed from the water bath and its ends were fixed onto two high-precision translation stages. One of which was driven by an arbitrary waveform generator (AWG), stretching the fibre in a sinusoidal fashion with amplitudes varying from  ± 200 n*ε* up to  ± 10 *με* and at frequencies up to 10 Hz. The setup modifications are shown in Fig. 5a. A sequence of R-OPO pulses mixed with the PM-shifted reference were captured over 3 s when initially stretching the fibre by  ± 2 *με* at a frequency of 2 Hz. The stacked FFT of captured pulses is displayed in Fig. 5b, where a strain coefficient of 0.131 GHz/*με* is observed.

At this point, it is worth mentioning the efficacy and simplicity of the R-OPO as a dynamic fibre sensor. Most distributed fibre sensors require complex and time-consuming algorithms to recover the applied strain. For instance, in Φ-OTDR techniques, complex phase recovery algorithms have been proposed to overcome problems such as intensity fading and phase unwrapping errors^35–38^. In chirped-pulse Φ-OTDR, strain variations are obtained by performing a correlation operation between sequentially captured traces, often requiring elaborate processing algorithms to mitigate accumulated errors^39^. The high computational demand of such processing algorithms makes them impractical for real-time strain sensing, usually limited to post-processing analysis. On the other hand, as shown in Fig. 5b, the R-OPO fibre sensor requires only a single FFT operation for complete strain recovery. Since the pulse waveform spreads over 10 ns, its FFT represents a 10 ns-screenshot of the refractive index state along the addressed fibre section. Moreover, since the time it takes to perform an FFT operation is much shorter than the interval between two pulses ( ~ 250 *μ*s), the R-OPO sensor enables the observation of the fibre state in real-time, without missing any pulses for the purpose of signal processing. Therefore, the system is free from complex and computational intensive algorithms, offering a fast and straightforward solution for fibre sensing applications. In fact, off-the-shelf oscilloscopes allow direct FFT calculation of captured traces, enabling real-time observation of the beat frequency variation (see Supplementary Video), while some newer models come with a spectrogram option, which would trace the exact result shown in Fig. 5b.

Reducing the applied strain amplitude to the minimal value accepted by the translation stage of  ± 200 n*ε*, a new sequence of R-OPO pulses was captured. Their stacked FFT is shown in Fig. 5c, with the inset displaying a zoom in the vertical axis. A variation of 200 n*ε* corresponds to a frequency shift of 26.2 MHz, which, even though is less than the spectral width of the beat frequency tone, it can still be easily observed as a sinusoidal shift of the beating tone peak frequency. When increasing the strain amplitude to  ± 10 *με*, the beat frequency tone exhibits strong frequency jumps as shown in Fig. 5d, governed by mode-hopping in the R-OPO. The same frequency-unwrapping algorithm used before to extend the dynamic measurement range for temperature sensing is here employed, resulting in the unwrapped beat frequency plot shown in Fig. 5e, accurately recovering the applied strain. Despite the accurate strain recovery, the dynamic measurement range cannot be arbitrarily increased: in case of a high amplitude and high frequency strain variation, the repetition rate of R-OPO pulses may not be enough to sample beat frequency jumps larger than Δ*ν*_*B*_ (equivalent to phase jumps larger than *π* in conventional phase unwrapping techniques). In order to test the R-OPO sensor response to higher strain frequencies, the AWG frequency was increased to 10 Hz while setting the strain amplitude to  ± 2 *με*. The experimental result is presented in Fig. 5f, showing the correct recover of the applied strain.

The generation of dynamic strain above 10 Hz was limited by the available instrumentation. For low strain amplitudes, when operation is mode-hop free, the detection system is limited by the Nyquist criterion, so that vibration frequencies up to 2 kHz could be detected for a pulse repetition rate of 4 kHz. Under mode-hopping, the maximum detection frequency is further limited. Since the sensor is an optical *oscillator*, whenever mode-hopping occurs, a dwell-time of several round-trips is required to reach the oscillation threshold. By changing the pulse generator configuration (see Fig. 2a) to send a burst of 100 pulses, it was possible to analyse the power growth of R-OPO pulses in time domain. The pulse peak power typically grows up to the 20th pulse, when it saturates after reaching the gain clamping point. Hence, considering a pulse period of 250 *μ*s (4 kHz), then a dwell-time of 5 ms is required for a detection with high SNR, limiting the maximum strain frequency to approximately 200 Hz.

### Increasing R-OPO sensitivity

**Fig. 6:**
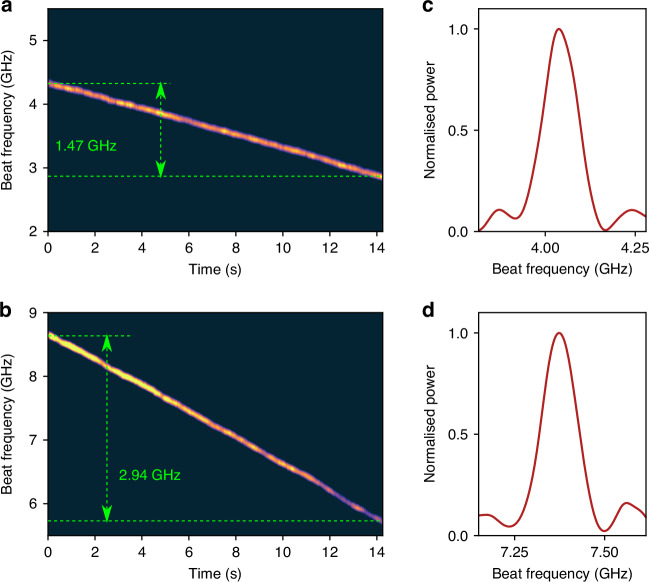
**Enhancing the intrinsic sensitivity**. Exploring the available FWM by-products, the sensitivity is enhanced from 1.33 GHz/°C (**a**) to 2.66 GHz/°C (**b**), resulting in a frequency shift twice as large for the same temperature change. The temperature accuracy remains unchanged since the spectral width is governed by the pulses’ Fourier transform: (**c**, **d**) show examples of spectra taken from (**a**, **b**), respectively

The intrinsic sensitivities of 0.131 GHz/*με* and 1.33 GHz/°C obtained before are determined by the refractive index’s dependence on temperature and strain in the sensing fibre. Due to the availability of high-order FWM by-products resulting from the interaction between the R-OPO oscillating line and the pump wavelength (see Fig. 2c and ref. ^23^), shifts in the R-OPO oscillating line result in *N* times shifts of the *N*^th^ order FWM by-product. Thus, this can be explored to significantly enhance the R-OPO sensitivity to strain or temperature changes. This was verified with temperature measurements by splitting the R-OPO output at port B in two branches and using two OBPFs, one still selecting the R-OPO oscillating line around 1550.28 nm and serving as a temperature reference, and the second filtering the 2^nd^ order FWM by-product at 1550.43 nm (see Fig. 2c). The novel selected spectral line beats with the fourth sideband of the pump laser light shifted by the PM, which had its driving frequency increased to 13.5 GHz. Two photodetectors were used while the temperature of the water bath increased by 1.1 °C. The stacked spectra of detected pulses are shown in Fig. 6a and b for the R-OPO oscillating line and the FWM by-product, respectively. The beat frequency shift of 2.94 GHz observed in Fig. 6b is twice of that shown in Fig. 6a, confirming a sensitivity improvement of a factor two.

If not limited by the SNRs of the FWM by-product and the shifted reference light, even higher sensitivity improvements could be obtained. One could argue that larger improvements may come at the cost of reduced measurement accuracy, which is related to the spectral width of the detected beat frequency signal. Nevertheless, since the linewidth of R-OPO is in the order of a few kHz^23^, and that of the PM-shifted reference light is governed by the pump laser linewidth (5 kHz), the spectral width of the detected beating tone is rather defined by the Fourier transform of 10 ns pulses, i.e., around 100 MHz. Thus, the measurement accuracy is not affected by choosing higher-order FWM by-products in the present case. This is verified in Fig. 6c, d, showing two examples of spectra with approximately the same width selected from Fig. 6a and b, respectively.

### Noise-limited sensitivity

**Fig. 7:**
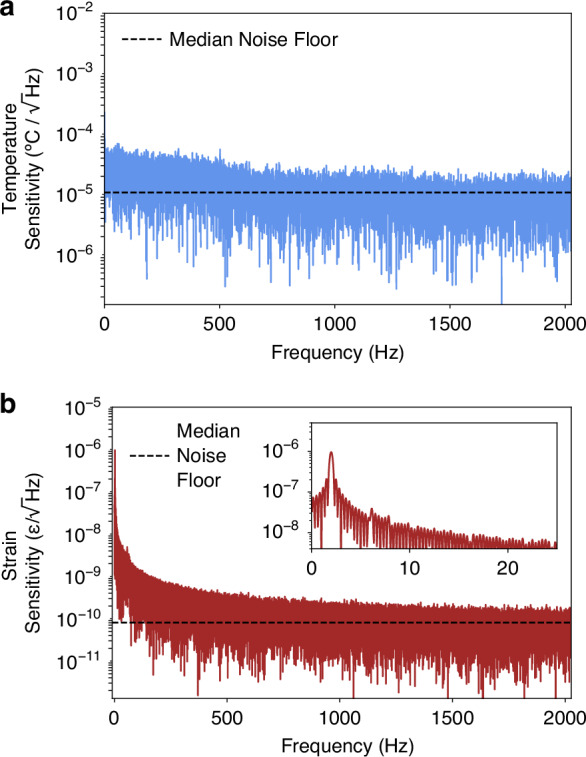
**Noise spectral densities**.(**a**, **b**) present the amplitude spectral densities of temperature and strain results, respectively. The noise-limited sensitivities obtained were 10.73 ${\mu }^{o}C/\sqrt{Hz}$ and 80.6 $p\varepsilon /\sqrt{Hz}$, calculated from the median noise floor

The temperature and strain noise-limited sensitivities were calculated from the noise spectral densities of the peak beat frequency measured over time under external perturbations. Temperature noise-limited sensitivity was derived from the first 3 s of the result shown in Fig. 3d, when temperature was linearly increased and the R-OPO was mode-hop free. The power spectral density result is shown in Fig. 7a, indicating a noise-limited sensitivity of 10.73 ${\mu }^{o}C/\sqrt{Hz}$. The strain sensitivity was derived from an applied strain of  ± 2 *με* at 2 Hz also over 3 s, just as shown in Fig. 5c. The result is displayed in Fig. 7b, where a peak tone of 2 Hz is observed (inset), and a noise-limited sensitivity of 80.6 $p\varepsilon /\sqrt{Hz}$ was obtained. Compared to a low-noise fibre sensor recently proposed^40^, enabling noise-limited sensitivities of 540 ${\mu }^{o}{\rm{C}}/\sqrt{Hz}$ and 4500 $p\varepsilon /\sqrt{Hz}$, the R-OPO sensitivity is significantly superior. Such low noise spectral density comes from the oscillatory behaviour of the R-OPO sensor. The requirement of multiple round-trips to achieve a laser-like oscillation naturally selects a single and well-defined optical frequency, which, in our proposed technique, is directly recovered from a single FFT operation, making it immune to calculation errors such as in phase unwrapping algorithms required in conventional Φ-OTDR.

Although the noise-limited sensitivities obtained are a good performance indicator, they do not correspond to the state-of-the-art values among the diverse fibre sensors reported. For instance, a world-class detection limit of 560 $f\varepsilon /\sqrt{Hz}$ have been recently reported by using a dual-comb distributed acoustic sensor (DAS)^41^. However, the R-OPO sensor still stands out due to its unique set of capabilities that are not offered by other techniques, including forward-transmission sensing, high SNR due to the laser-like oscillation, enhanced intrinsic sensitivity, flexible single location sensing, and straight-forward setup and processing technique.

### Alternative sensing setup

**Fig. 8:**
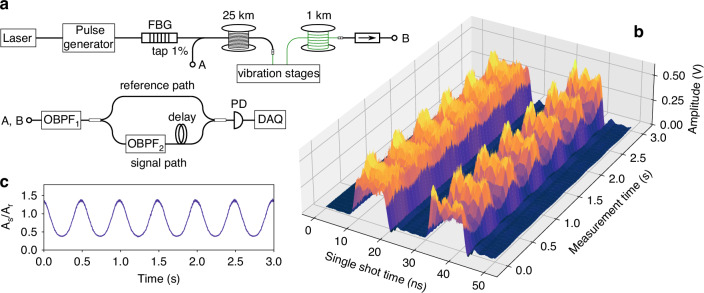
**Low-bandwidth detection setup**. **a** An alternative setup is proposed for the detection of R-OPO pulses with low-bandwidth components. **b** Two optical pulses arrive in a low-bandwidth photodetector (PD) in sequence, first a reference pulse, then a signal pulse. **c** By calibrating the intensity of signal pulses with that measured for reference pulses, a sinusoidal strain pattern is observed, matching with the applied strain variation

The experimental setup depicted in Fig. 2a provides a simple way to recover environmental perturbations applied to a selected fibre segment. Even though the data processing step is straightforward, the setup requires high-frequency bandwidth components: a 12 GHz RF synthesizer was employed to shift the reference light, and high-bandwidths PD, PM and oscilloscope were needed. In this section, we propose an alternative detection setup, offering similar sensing capabilities but demanding only low-frequency components. The principle is similar to that presented in^42^, but it is further improved here where only a single PD is needed. The setup is shown in Fig. 8a. The R-OPO is prepared in the same way as before, while the detection scheme is replaced with a dual pulse configuration. The R-OPO pulses tapped either at point *A* or *B* are first filtered with an OBPF (OBPF_1_) to select the the R-OPO oscillating line, and then split into two paths, a reference path and a signal path. Light from the signal path passes through a second OBPF (OBPF_2_), which is tuned to have one of its spectral edges aligned with *ν*_*B*_. Hence, any spectral shift of the R-OPO oscillating line will cause an intensity change on the pulses travelling on the signal path. A fibre delay line of 4 m (20 ns) is added to the signal path so that pulses from the reference and signal paths do not overlap in time, enabling the detection of both pulses with a single low-bandwidth PD (200 MHz) and data acquisition card (DAQ). Pulses from the reference path are used to calibrate intensity changes measured at the pulses travelling through the signal path. Similar to the jagged trace of Φ-OTDR systems, where changes of the refractive index at a given fibre segment imparts on a non-linear intensity change of the detected trace, here, environmental perturbations not only cause a shift in the R-OPO oscillating frequency, but also affects the envelope of R-OPO pulses in a non-linear way. Thereby, reference pulses are used to discount these intensity changes from the signal pulses. Frequency shifts of the R-OPO are thus calculated from the following ratio:

7 $$\Delta f=\gamma \,\frac{{A}_{s}}{{A}_{r}}=\gamma \,\frac{\int\,s(t)dt}{\int\,r(t)dt}$$

where *A*_*s*_ and *A*_*r*_ represent the energy of signal and reference pulses, respectively, both calculated from the integral of pulses waveforms (*s*(*t*), signal and *r*(*t*), reference). The proportionality constant *γ* depends on the slope of OBPF_2_’s edge.

A sinusoidal strain of  ± 2 *με* at a frequency of 2 Hz was applied to the addressed fibre segment and the pair of reference/signal pulses was continuously detected over 3 s. The experimental result is shown in Fig. 8b. Clearly, both the signal and reference pulses are affected by the applied strain, where a stronger intensity change is observed for signal pulses (arriving later, from 30 to 40 ns in Fig. 8b). When using Eq. (7) for frequency-shift recovery, a sinusoidal pattern is obtained as shown in Fig. 8c. It is worth noting that the linearity between Δ*f* and *A*_*s*_/*A*_*r*_ assumed in Eq. (7) actually depends on the spectral shape of the edge of OBPF_2_. In case the filter’s edge is not linear across the FBG’s bandwidth ( ~3 GHz), then a non-linear conversion would be obtained. Nevertheless, the possibility of a long distance, low-cost, low-bandwidth, high SNR and high spatial resolution fibre sensing scheme turns to be an interesting option for practical implementation.

## Discussion

Unlike other pulsed-based fibre sensors, which must operate under low pulse powers to prevent non-linear effects, the R-OPO fibre sensor takes advantage of non-linear effects to allow long-distance-access sensing, offering a sensing range of 1 km that is located beyond 25 km from the instrumentation. In our experiments, the sensing range was limited by the available sensing fibre length; however, longer fibres could be used, provided that the sensing fibre remains shorter than the gain fibre. It is worth noting that no special properties are required for the gain fibre, so that it can be used to access the sensing region. In case this region was located 50 km apart from the detection instrumentation, a 50 km-long launching fibre connecting the instrumentation to the sensing fibre would act as gain medium. Note that, as the non-linear effective distance is limited to approximately 22 km, the last 28 km of the launching fibre would primarily introduce additional loss. However, if sufficient MI gain is available to compensate the resulting total cavity loss of 66 dB, then random parametric oscillations would initiate enabling the sensing of environmental perturbations. This could be achieved if higher pulse powers were used, provided that other non-linear effects were suppressed in favour of MI^43^. Due to the inherent characteristics of the proposed laser-like sensor, high SNR ( > 15 dB) and high spatial resolution ( < 1 m) are maintained even at ultra-long distances.

A spatial resolution of 1 m was achieved in this work, demonstrating the capability of the system. In principle, even finer spatial resolutions could be achieved if sufficient gain is available. The pump pulses width at the same time defines the spatial resolution and governs the loss level in the parametric oscillator (see Eq. (2)), with shorter pulses requiring higher gain to achieve oscillation. One possible approach to improving the spatial resolution involves the use of hybrid gain mechanisms^20^; by combining MI gain with an additional amplification process, it may be possible to employ shorter pulses. Note that, although the gain fibre does not require special properties, the system has so far only been demonstrated using a sensing fibre with enhanced reflectivity. Nonetheless, the combination of hybrid gain mechanisms with broader pump pulses (compromising the spatial resolution) could open the possibility of operating the system entirely within standard single-mode fibre (SMF). This remains an avenue for future investigation.

A notable aspect observed during the experiments was that just above the parametric oscillation threshold, minor polarization variations imposed by manually changing the state of polarization of pump pulses could interrupt parametric oscillation. This is a result of polarization-dependent gain in fibre-optic parametric amplifiers^44^; misaligning the polarization of pump pulses and reflected seed light traduces into additional losses in the cavity. To mitigate this problem, the polarization of pump pulses was first aligned with that of reflected light from the addressed fibre section, and all experiments were conducted at a pump power sufficiently above the threshold, ensuring robustness against polarization drifts. In addition, it is important to note that sensing information in the R-OPO system is encoded in the spectrum of output pulses rather than their intensity. Therefore, as long as oscillation is sustained, polarization-dependent gain/noise does not impact the sensing performance. Nonetheless, to maintain optimal oscillation conditions, the average intensity of detected pulses could serve as a feedback signal for a polarization control system employing a polarization tracker, thereby preserving alignment between the pump and reflected signal polarizations.

The convenience of single-end access, as well as the dynamic sensing capabilities of the R-OPO fibre sensor makes it a unique EAFS. Another major advantage of the R-OPO is that the sensing signal is available at both ends of the fibre, thus enabling forward transmission sensing^45^. Although many works have successfully measured vibrations in fibres by monitoring fast changes in the transmitted phase or polarization, most of them fail to identify the vibration location. The R-OPO sensor enables effortless forward sensing: each detected pulse carries information of the sensing quantity (temperature/strain changes) on its intra-pulse frequency (order of GHz), while the pulse repetition rate provides the sensing location (order of kHz).

We demonstrated that temperature and strain variations can be readily extracted using the proposed sensor through a single FFT operation, enabling real-time sensing without the need for complex post-processing algorithms. Moreover, the artefact of FWM by-products could be effectively leveraged to enhance the measurement sensitivity. In addition, through a simple frequency-unwrapping algorithm we significantly enhanced the dynamic measurement range of temperature/strain changes. Due to the inherent light oscillation properties of the R-OPO, remarkably low noise-limited sensitivities of 10.73 ${\mu }^{o}C/\sqrt{Hz}$ and 80.6 $p\varepsilon /\sqrt{Hz}$ were obtained for temperature and strain sensing, respectively. Therefore, we expect the R-OPO fibre sensor to stand out in single-end long-distance-access fibre sensing applications, where the sensed location can be accurately tuned by simply changing the repetition rate of pump pulses.

## Materials and Methods

### Pulse generator

Pump pulses with high extinction-ratio were prepared in the same way as described in^23^: light from a continuous wave semiconductor laser operating at 1550.05 nm was directed to two electro-optic modulators (EOMs) driven by an AWG with two channels. Square pulses with 10 ns-duration were set in each channel to drive the EOMs, with an electronic delay added to the pulse driving the second EOM to compensate for the fibred optical path between the EOMs. To achieve high pump powers, two amplification stages were used, each composed of a high-power EDFA and an OBPF, the latter used to suppress amplified spontaneous emission noise (ASE).

### Frequency-unwrapping algorithm

The developed frequency-unwrapping algorithm, designed to extend the dynamic measurement range, operates as follows: it detects discontinuities in the peak frequency of the measured spectrogram (see example in Fig. 4a) and corrects them to ensure a continuous representation of external perturbations. The main steps of the method are shown in Algorithm 1.

Let *N* be the number of detected pulses during an arbitrary measurement time. The spectrum of the *i*^th^ detected pulse can be written as *S*(*i*, *f*), where *i* ∈ [1, *N*]. Discontinuities in the peak frequency are identified when the difference between subsequent peak frequencies are beyond a predetermined threshold, Δ*f*_th_, which we set to 0.1 GHz. Certainly, external perturbations causing frequency shifts greater than Δ*f*_th_ between subsequent pulses would be considered the result of mode-hopping, and thus wrongfully corrected by the algorithm. This limitation is inherent to unwrapping algorithms, such as in phase-unwrapping, where phase shifts greater than 2*π* induce an unwrapping error. However, for sufficiently high sampling rates, i.e., pump pulse repetition rates, frequency-unwrapping errors are completely hindered.

After setting Δ*f*_th_, an accumulated correction of frequency shifts Δ*f*_acc_ is initialized as zero. The peak frequency of the first detected spectrum *S*(1, *f*) is assigned to *f*_peak_, calculated with a straightforward peak detection algorithm *getPeakFreq*. Then, we loop through all detected spectra while comparing the difference between subsequent peak frequencies (*f*_diff_ = *f*_peak_ − *f*_0_), with Δ*f*_th_. In case a discontinuity is identified, then the unwrapped spectrogram *S*_unw_ is corrected by the accumulated frequency shift Δ*f*_acc_.

#### Algorithm 1

Frequency-unwrapping

 1: **Set:** Δ*f*_th_ ← 0.1 GHz

 2: **Initialize:** Δ*f*_acc_ ← 0

 3: **Initialize:** *f*_peak_ ← *getPeakFreq*(*S*(1, *f*))

 4: **for** *i* ← 2 to *N* **do**

 5:  *f*_0_ ← *f*_peak_

 6:   *f*_peak_ ← *getPeakFreq*(*S*(*i*, *f*))

 7:   *f*_diff_ ← *f*_peak_ − *f*_0_

 8:   **if** ∣*f*_diff_∣ > Δ*f*_th_ **then**

 9:    **if** *f*_diff_ > 0 **then**

10:     Δ*f*_acc_ ← Δ*f*_acc_ − *f*_diff_

11:    **else**

12:      Δ*f*_acc_ ← Δ*f*_acc_ + *f*_diff_

13:    **end if**

14:   **end if**

15:   *S*_unw_(*i*, *f*) ← *S*(*i*, *f* + Δ*f*_acc_)

16: **end for**

## Supplementary information

Supplemental Material

Supplementary Video

## Data Availability

The data that support the findings of this study are available from the corresponding author upon reasonable request.
