# Supplementary material for: Random optical parametric oscillator fibre sensor
Source: Light Sci Appl. 2026 Jan 4;15:52. doi: 10.1038/s41377-025-02049-9 (PMC12764772; doi:10.1038/s41377-025-02049-9)
Supplement: Supplementary file 1 — Supplemental Material [file 41377_2025_2049_MOESM1_ESM.pdf]

Supplementary Information for:

Random Optical Parametric Oscillator Fibre Sensor

Authors

Pedro Tovar[1,*]
Jean Pierre von der Weid[2]
Yuan Wang[1]
Liang Chen[1]
Xiaoyi Bao[1]

Affiliations

[1] Nexus for Quantum Technologies, University of Ottawa, Ottawa, Ontario K1N 6N5, Canada
[2] Department of Electrical Engineering, Pontifical Catholic University of Rio de Janeiro, 22451-900, Rio de Janeiro, RJ, Brazil
[*] Corresponding author email: ptovar@opto.cetuc.puc-rio.br

Supplementary Notes

Modulation instability (MI) is a well-know non-linear phenomenon present in dispersive media, which has been observed in many fields of study, such as fluid dynamics, plasma physics and optical fibres [1]. In the latter, it occurs when a strong optical wave breaks into a sequence of soliton-like pulses, driven by tiny residual modulations originated from intensity fluctuations. MI is enabled by an interplay between the Kerr effect and dispersion, and it can be predicted by applying a linear stability analysis to the non-linear Schröedinger equation (NLSE) [1]:

$$i\frac{\partial A}{\partial z} - \frac{\beta_2}{2}\frac{\partial^2 A}{\partial \tau^2} + i\frac{\alpha}{2}A + \gamma P_0 |A|^2 A = 0 \tag{1}$$

where $A(z,t)$ is the slowly varying envelope normalized to the input power P_0, travelling with group velocity $v_g = 1/\beta_1$. The fibre's non-linear coefficient is γ, the group velocity dispersion coefficient is β_2, and α is the attenuation coefficient.

Many approaches have been proposed over the years to solve Eq. 1 numerically, for instance the split-step Fourier method (SSFM) [2] and finite-difference method [3]. In the MI framework, an analytical model has been proposed and experimentally validated in [4]. Authors have shown that model predictions match experimental observations under the low-depletion regime. Based on a signal-idler approach and applying linear stability analysis, the MI-gain spectrum can be expressed as [4]:

$$G_{\mathrm{MI}}(\Omega) = e^{-\alpha L}\left(1 + 2\left(\frac{\gamma P_0}{g(\Omega)}\right)^2 \sinh^2(g(\Omega)L_{\mathrm{eff}}\right) \tag{2}$$

where L_{eff} is the fibre effective length given by $L_{\mathrm{eff}} = (1 - e^{-\alpha L})/\alpha$. The frequency-dependent parametric gain coefficient $g(\Omega)$ is defined as:

$$g(\Omega)^2 = -(\beta_2\Omega^2)\left(\gamma P_0 + \frac{\beta_2\Omega^2}{4}\right) \tag{3}$$

where Ω represents the frequency detuning around the pump frequency ω_0, such that $\Omega = \omega - \omega_0$.

Eqs. 2-3 can be used to simulate the propagation of MI-gain spectrum along single-mode fibres (SMF). We considered the following typical values for standard SMFs in our simulations: $\alpha = 0.2$ dB km^{-1}, $\beta_2 = -22$ ps^2 km^{-1}, and $\gamma = 1.8$ W^{-1} km^{-1}. We calculated the distributed MI-gain spectrum along a fiber length of 25.5 km, matching the one used in our experimental setup, and an input peak power of 285 mW. The theoretical result is shown in Figure S1(a), which clearly depicts the build-up of MI-gain along the fibre. In Figure S1(b), the accumulated gain spectrum at position 25.5 km is presented, exhibiting a peak MI-gain of 58.7 dB at a detuning frequency of $\sim$30 GHz. As shown in the main text, this gain is sufficient to compensate for the round-trip losses, thus enabling the onset of parametric oscillation.

Supplementary Video Note

To show the real-time capabilities of the R-OPO fibre sensor, we recorded the screen of an off-the-shelf oscilloscope (Keysight MSOV334A) while applying a sinusoidal strain of $\pm 2\ \mu\varepsilon$ at a frequency of 0.5 Hz. In the recorded video (provided with this submission), the oscilloscope screen is split vertically into two channels. The top channel shows the detected R-OPO pulse mixed with the reference light (see Fig. 5a in the main text). The bottom channel shows the FFT operation performed on the top channel. A spectral peak is observed around 5 GHz, representing the beating between the R-OPO pulses and the reference light. The peak shifts sinusoidally with the applied strain, which is readily observed in the oscilloscope, where no complex post-processing techniques is required. Since frequency shifts are directly proportional to strain variations, the R-OPO fibre sensor offers real-time quantification of strain/temperature changes.

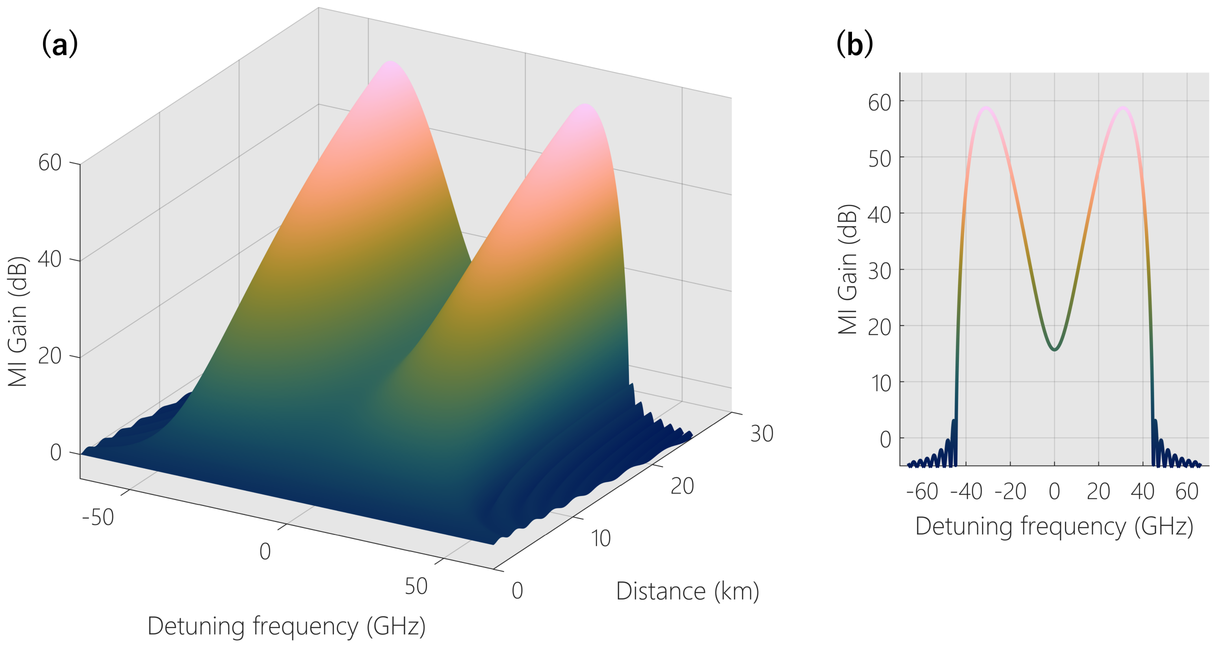

Figure S1: **Distributed Modulation Instability gain spectrum**. **a** Theoretical MI-gain spectrum calculated when launching pump pulses with a peak power of 285 mW along 25.5 km of standard single-mode fibre with the following typical parameters: $\alpha = 0.2$ dB km^{-1}, $\beta_2 = -22$ ps^2 km^{-1}, and $\gamma = 1.8$ W^{-1} km^{-1}. **b** MI-gain spectrum at 25.5 km.

References

[1] G. Agrawal, *Nonlinear Fiber Optics*. Optics and Photonics, Elsevier Science, 2012.

[2] R. A. Fisher and W. K. Bischel, "Numerical studies of the interplay between self-phase modulation and dispersion for intense plane-wave laser pulses," *Journal of Applied Physics*, vol. 46, pp. 4921–4934, 11 1975.

[3] Q. Chang, E. Jia, and W. Sun, "Difference Schemes for Solving the Generalized Nonlinear Schrödinger Equation," *Journal of Computational Physics*, vol. 148, no. 2, pp. 397–415, 1999.

[4] Mehdi Alem and Marcelo A. Soto and Luc Thévenaz, "Analytical model and experimental verification of the critical power for modulation instability in optical fibers," *Opt. Express*, vol. 23, pp. 29514–29532, Nov 2015.
